# Identification of a mitochondrial-binding site on the N-terminal end of hexokinase II

**DOI:** 10.1042/BSR20150047

**Published:** 2015-06-12

**Authors:** Nadezda Bryan, Kevin P. Raisch

**Affiliations:** * Department of Otolaryngology, University of Florida, Gainesville, FL 32610, USA

**Keywords:** cancer, glycolysis, hexokinase II, mintochondrial-binding site, mutations

## Abstract

Hexokinase II (HKII) is responsible for the first step in the glycolysis pathway by adding a phosphate on to the glucose molecule so it can proceed down the pathway to produce the energy for continuous cancer cell growth. Tumour cells overexpress the HKII enzyme. In fact, it is the overexpression of the HKII enzyme that makes the diagnosis of cancer possible when imaged by positron emission tomography (PET). HKII binds to the voltage-dependent anion channel (VDAC) located on the mitochondrial outer membrane (MOM). When bound to the MOM, HKII is blocking a major cell death pathway. Thus, HKII is responsible for two characteristics of cancer cells, rapid tumour growth and inability of cancer cells to undergo apoptosis. One method to identify novel compounds that may interfere with the HKII–VDAC-binding site is to create a molecular model using the crystal structure of HKII. However, the amino acid(s) responsible for HKII binding to VDAC are not known. Therefore, a series of truncations and point mutations were made to the N-terminal end of HKII to identify the binding site to VDAC. Deletions of the first 10 and 20 amino acids indicated that important amino acid(s) for binding were located within the first 10 amino acids. Next, a series of point mutations were made within the first 10 amino acids. It is clear from the immunofluorescence images and immunoblot results that mutating the fifth amino acid from histidine to proline completely abolished binding to the MOM.

## Abstract (precis)

The first ten amino acids of hexokinase II are import for binding to the MOM. Mutating the fifth amino acid abolished mitochondrial binding. Determining the binding site is important for identifying novel compounds that can disrupt HKII binding to VDAC.

## INTRODUCTION

In 1931 Dr Otto Warburg won the Nobel Prize in Physiology and Medicine for his discoveries associated with respiratory enzymes. Dr Warburg's early studies, known as the Warburg effect, were the first to describe the high rate of glucose uptake by tumour cells with an increased release of lactic acid [1,2]. Dr Warburg hypothesized that tumour cell growth is caused by a defect in the mitochondria leading to an increased reliance on the breakdown of glucose (glycolysis) to generate ATP [3]. Subsequently, studies have shown that tumour cells overexpress hexokinase II (HKII) and the overexpression of HKII plays a critical role in maintaining the high rate of glycolysis in tumour cells. HKII has a direct relationship with two major hallmarks of tumour cells: (1) the increased rate of glycolysis and subsequent ATP production and (2) the decreased ability to undergo apoptosis. HKII binds to the voltage-dependent anion channel (VDAC) on the mitochondrial outer membrane (MOM), thereby blocking BAX (BCL2-associated X protein) from activating the mitochondrial-related cell death pathway.

Subcellular studies have shown that HKII is catalytically active when bound to the MOM [4,5]. Agents that induce the translocation of HKII from the MOM to the cytoplasm have been shown to disrupt the glycolysis pathway and sensitize tumour cells to chemotherapy drugs [6–8]. Our laboratory has shown that the translocation of HKII from the MOM to the cytoplasm arrests the cells in the late G_1_-cell cycle phase and thereby sensitizes tumour cells to radiation treatment [9].

The goal of the present study was to identify the amino acid(s) in HKII that are associated with binding to VDAC on the MOM. The binding site to VDAC has been reported to be within the first 15–23 amino acids of the N-terminal end [10–12]. Therefore, a series of truncations and point mutations were made to identify the amino acids critical for binding to VDAC. Deletions of the first 10 and 20 amino acids indicated that important amino acids for binding to the MOM were located within the first 10 amino acids. A series of single point mutations were created within the first 10 amino acids of the N-terminal end of HKII. All HKII proteins were tagged with green fluorescent protein (GFP) in order to follow their expression and localization by fluorescence microscopy. Immunoblot analyses were used on mitochondrial and cytoplasmic fractions to confirm the microscopic observations presented herein.

## EXPERIMENTAL

### Mutating hexokinase II and plasmid constructs

Deletions and point mutations were made using the HKII eukaryotic expression vector, pFLHKII-pGFP-N3, developed by Dr Ardehali and purchased from Addgene as the template for PCR-based mutations [13]. All PCR primers were synthesized by Eurofins Genomics. Since the VDAC-binding site was predicted to be within the first 20 amino acids of the N-terminal end of HKII, two deletion mutants of HKII were initially designed to narrow the region for point mutations (Table 1). Deletions of the first 10 (HKII pI2_F10del) and 20 (HKII pI2_Q20del) amino acids of the N-terminal end of HKII were cloned in-frame with GFP. The downstream PCR primers were designed to incorporate an EcoRI restriction enzyme site downstream of the start codon and an annealing region of 26 nucleotides upstream of the deleted region. The downstream primers were GCTAGCCTACGATTCGAATTCATGTTCACGGAGCTCAACCATGACCAAGT (pI2_F10del) and GCTAGCCTACGATTCGAATTCATGAAGGTTGACCAGTA-TCTCTACCACAT (pI2_Q20del).

**Table 1:**
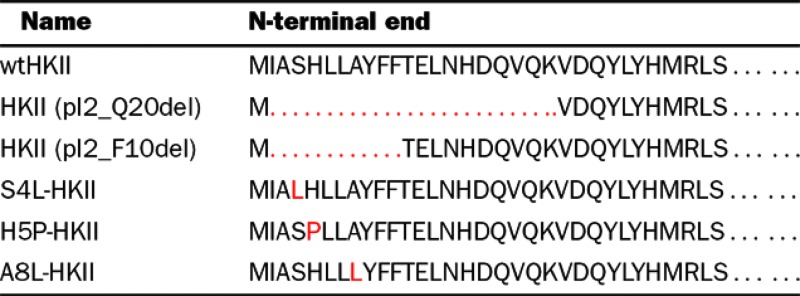
Hexokinase II mutations were made in the putative VDAC-binding site located on the N-terminal end Deletion mutations of 20 amino acids (pI2_Q20del) and 10 amino acids (pI2_F10del) by PCR and the clones analysed for binding to the mitochondrial membrane. Using PredictProtein [14,15], 3 amino acids were predicted to bind protein within the 10 amino acid deletion. Single point mutations at amino acids 4(S4L), 5(H5P) and 8(A8L) were made by PCR and the clones analysed for binding to the mitochondrial membrane. Mutations depicted in red.

Using the ISIS method in PredictProtein (https://www.predictprotein.org), 3 amino acids were predicted to bind proteins within the first 10 amino acids of HKII [14,15]. The following downstream primers were synthesized to incorporate point mutations in the fourth amino acid, serine to leucine (S4L), fifth amino acid histidine to proline (H5P) and the eighth amino acid alanine to leucine (A8L): GCTAGCCTACGATTCGAATTCATGATTGCCTTGCATCT-GCTTGCCTACTTCTTCACGGAGCTAACCATGACCAAGT (S4L); GCTAGCCTACGATTCGAATTCATGATTGCCTCGC-CTCTGCTTGCCTACTTCTTCACGGAGCTCAACCATGA-CCAAGT (H5P); GCTAGCCTACGATTCGAATTCATGATT-GCCTCGCATCTGCTTCTCTACTTCTTCACGGAGCTCAA-CCATGACCAAGT (A8L). The upstream PCR primer (ATGG-TCCCAACTG-TGTCATT) incorporated an EcoRV restriction enzyme site and was used in all PCR amplifications. For each PCR, the reaction buffer consisted of 1.5 mM MgCl_2_, 0.2 mM each dNTP, 10 pmol/50 μl each upstream and downstream primer, 2 ng plasmid DNA and 2.5 units GoTaq polymerase (Promega). The reaction conditions for all primer sets were 95°C for 2 min followed by 35 cycles of 95°C for 30 s, 55°C for 1 min, 72°C for 1 min with a final 5 min extension at 72°C. All PCR amplicons were separated using 1% agarose gel electrophoresis, stained with ethidium bromide and detected by UV light using a VersaDoc 1000 camera system (Bio-Rad Laboratories).

Amplicons with an expected size of approximately 600 bp were excised from the agarose gel using X-tractor gel extractor tool (Promega) and eluted from the gel using Wizard SV Gel and PCR Clean-Up System (Promega). PCR amplicons and pFLHKII-pGFP-N3 were cut with the restriction enzyme, EcoRI (Promega), for 1 h at 37°C, then heat inactivated and precipitated after adding one-tenth volume 5 M NaCl, 2 volumes ethanol and 1 μg glycogen as a carrier. The EcoRI cut amplicons and plasmid was suspended in water then cut with the restriction enzyme, EcoRV (Promega), for 1 h at 37°C. Following heat-inactivation, the double-digested plasmid was separated by 1% agarose gel electrophoresis and eluted from the gel as described above. The double-digested amplicons and plasmid were precipitated as described above. Double-digested plasmid DNA was suspended in water and reacted with calf intestinal alkaline phosphatase (CIAP, Promega) as described by the manufacturer's protocol then heat inactivated and the DNA precipitated with glycogen as a carrier. Each amplicon and plasmid was suspended in water, then the amplicons were ligated to the plasmid DNA using T4 DNA ligase (Promega) for 18 h at 4°C using a vector:insert ratio of 3:1. Competent JM109 cells (Promega) were transformed with the ligated plasmid DNA and colonies were selected on LB agar plates containing kanamycin (50 μg/ml). Following a 24 h incubation at 37°C, individual colonies were selected and grown overnight in LB broth with kanamycin at 37°C. Plasmid DNA was isolated from the overnight broth cultures using PureYield Plasmid miniprep system (Promega). Plasmid DNA was submitted for sequencing to Eurofins Genomics using primers upstream and downstream of the ligation sites. Sequencing results were analysed using MacVector Software v12.7.5 and plasmids containing the correct truncation or point mutation in-frame with HKII-GFP were used for transfection experiments.

### Transfection of U-2OS cells

U-2OS cells (A.T.C.C.) were transfected with eukaryotic expression vectors containing truncations and point mutations of HKII using FuGene HD (Promega) as described in the manufacturer's protocol. Forty-eight hours after transfection, G418 (500 μg/ml) was added to the culture medium and the cells were maintained in G418-containing culture medium for 4 weeks. To select for stably-transfected cell lines, the G418 was removed from the culture medium for 2–4 weeks. Then G418 was added back to the culture medium and the cells were passaged for an additional 4 weeks before removing G418 from the culture medium. The surviving cells lines were considered stably-transfected with the plasmid DNA and were used for analysis by fluorescent microscopy and immunoblot analysis.

### Detection of HKII protein by immunoblot and fluorescence microscopy

Stably-transfected cells were grown in 4-chamber slides (Thermo Scientific Nunc) until 60–80% confluence. Living cell fluorescent images were captured using an AMG EVOS FL Cell Imaging System with a Plan Fluorite 40×/0.65 objective.

Immunoblots were used to confirm the redistribution of HKII and mutated HKII proteins into the cytoplasm from the mitochondria. The enriched-mitochondrial fractions and cytoplasmic fractions were collected and processed as described previously [9]. Equal concentrations of protein were separated by 10% SDS/PAGE and transferred to Immobilon-P membrane (Millipore Corp.) by electroblotting. HKII, mitochondrial Hsp70 (mtHsp70), and β-actin were identified by immunoblotting using the following antibodies purchased from Thermo Scientific; anti-HKII, anti-mtHsp70, anti-β-actin, anti-mouse-HRP, anti-rabbit-HRP. Antibodies targeting β-actin and mtHsp70 were used as loading controls for cytoplasmic and mitochondrial fractions, respectively. Protein was detected by chemi-luminescent signalling (Pierce ECL) and the immunoblots were scanned and analysed by densitometry using UN-SCAN-IT software (Silk Scientific, Inc.). The densitometry results were expressed as a ratio of the intensity of HKII to that of β-actin or Hsp70 from the same source.

## RESULTS

### Analysis of HKII deletion mutations

For the initial analysis of HKII binding to the MOM, deletion mutants were cloned into a vector and expressed in U-2OS cells. On the N-terminal end of HKII, the first 10 (pI2_F10del) and first 20 (pI2_Q20del) amino acids were deleted following the start codon, Met (Table 1). The wild-type FLHKII (full-length HKII) protein and the deletion mutants were cloned in-frame with the GFP-tag to allow for observation and localization by fluorescence microscopy. For the wild-type FLHKII enzyme (Figure 1A) the fluorescence was observed on the mitochondrial-like structures located around the nuclei. For the two deletion mutants (Figures 1B and 1C), a diffuse fluorescence was observed throughout the cytoplasm indicating that the first 10 and first 20 amino acids were important for binding to the MOM. To confirm the results of the fluorescence microscopy, cytoplasmic- and mitochondrial-enriched fractions were collected from stably-transfected cell lines expressing wild-type HKII and truncated mutants of the HKII. Wild-type FLHKII was detected by immunoblot in the cytoplasmic- and mitochondrial-enriched fractions (Figure 1D). However, the HKII deletion mutant proteins, pI2_F10del and pI2_Q20del, were absent from the mitochondrial-enriched fractions and only detected in the cytoplasmic-enriched fraction. In the cytoplasmic fraction, a 1.7-fold increase in HKII pI2_F10del and a 3-fold decrease in HKII pI2_Q20del protein expression were found as compared with the levels of FLHKII.

**Figure 1:**
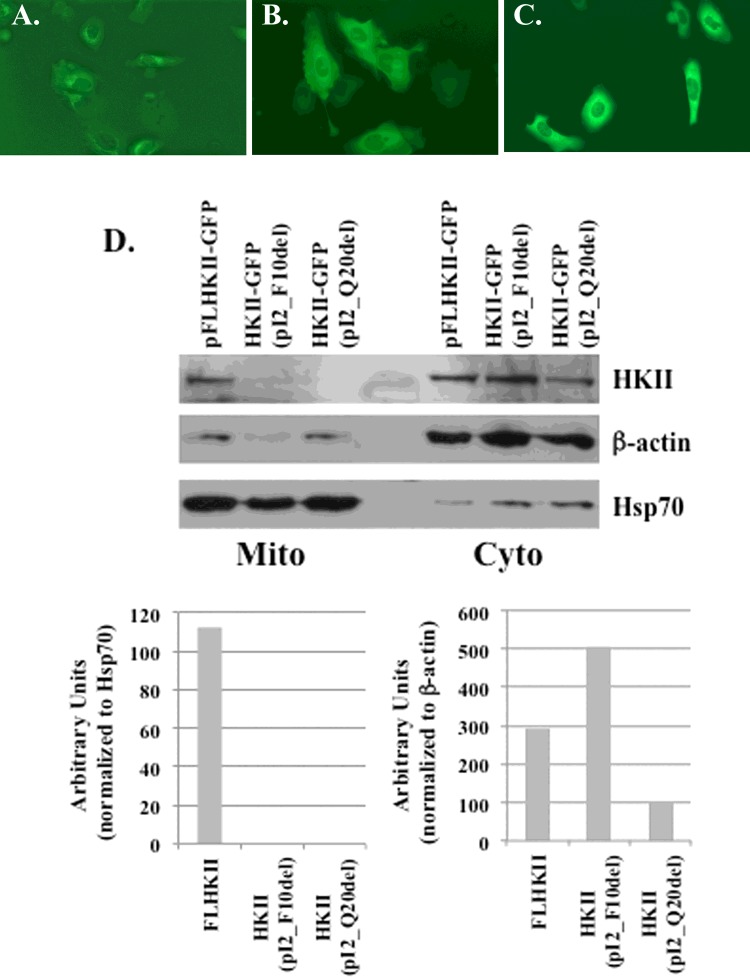
Fluorescent and immunoblot images of the HKII truncation mutants Stably-transfected U-2OS cells were grown in chamber slides with a plasmid carrying FLHKII, a 10aa deletion or a 20aa deletion in the N-terminal end of HKII. The cells were imaged at 400× magnification using an EVOS FL microscope. (**A**) U-2OS cells transfected with GFP-tagged wild-type FLHKII; (**B**) U-2OS cells transfected with GFP-tagged HKII (pI2_F10del); (**C**) U-2OS cells transfected with GFP-tagged HKII (pI2_Q20del); (**D**) immunoblot analysis of mitochondrial (Mito) and cytoplasmic (Cyto) fractions of U-2OS cells stably-transfected with wild-type FLHKII-GFP, HKII-GFP (pI2_F10del), or HKII-GFP (pI2_Q20del). Immunoblots were probed with primary antibodies to detect: hexokinase II, β-actin and mtHsp70. Densitometry was used to quantify the expression levels of HKII in the cytoplasmic and mitochondrial cell fractions.

**Figure 2:**
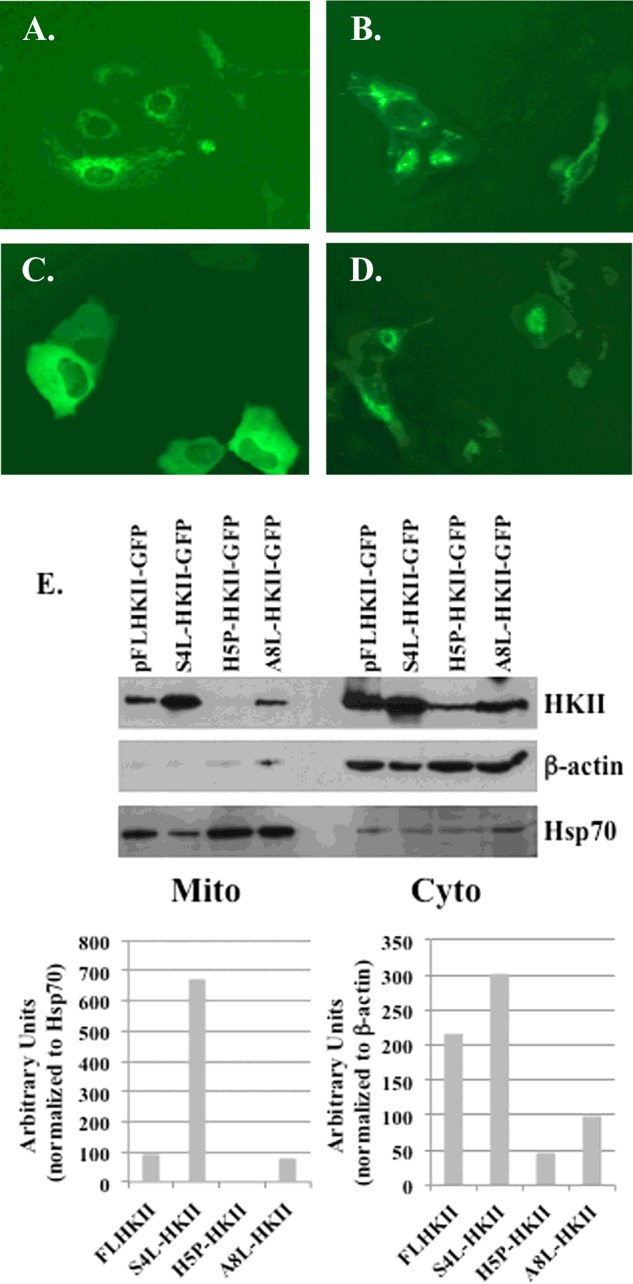
Fluorescent and immunoblot images of the HKII mutants with single point mutations U-2OS cells were transfected with plasmids carrying FLHKII or a point mutation of amino acids in position 4, 5, or 8. Stably-transfected cells were imaged at 400× magnification using an EVOS FL microscope. (**A**) U-2OS cells transfected with GFP-tagged wild-type FLHKII; (**B**) U-2OS cells transfected with (S4L)-HKII-GFP; (**C**) U-2OS cells transfected with (H5P)-HKII-GFP; (**D**) U-2OS cells transfected with (A8L)-HKII-GFP; (**E**) mitochondrial (Mito) and cytoplasmic (Cyto) fractions were isolated from U-2OS cells stably-transfected with wild-type FLHKII-GFP and each mutant HKII protein, then analysed by immunoblot. Immunoblots were probed with primary antibodies to detect: hexokinase II, β-actin and mtHsp70. Densitometry was used to quantify the expression levels of HKII in the cytoplasmic and mitochondrial cell fractions.

### Analysis of HKII point mutations

The results from the deletion mutations indicated that the amino acids required for binding to the MOM were located within the first 10 amino acids of the N-terminal end of HKII. Therefore the HKII amino acid sequence was submitted to PredictProtein and using the protein–protein interaction sites (ISIS) method, the fourth (serine), fifth (histidine), and eighth (alanine) amino acids within the first 10 amino acids were predicted to bind protein. Using PCR-based site-directed mutagenesis the serine and alanine were mutated to a leucine and the histidine was mutated to a proline (Table 1). The resultant transfected U-2OS cells lines were screened by fluorescence microscopy using the GFP-tag to visualize the location of the HKII proteins. The S4L-HKII and the A8L-HKII mutated proteins were found to maintain binding to mitochondrial-like membrane structures as observed with the wild-type FLHKII protein (Figures 2A–2D). Whereas, a diffuse fluorescence was observed in the cytoplasm of the cells expressing the H5P-HKII mutated protein (Figure 2C). To confirm the binding of HKII proteins to the MOM, mitochondrial-enriched and cytoplasmic-enriched fractions were isolated from stably-transfected cell lines. As shown in Figure 2E, the presence of HKII protein in the mitochondrial-enriched fractions was identified from cell lines expressing FLHKII, S4L-HKII and A8L-HKII protein but no HKII protein was detected from the H5P-HKII expressing cell line. A greater than 6-fold increase in S4L-HKII mutant protein was found in the mitochondrial fraction as compared with the wild-type FLHKII protein. In the cytoplasmic-enriched fractions, HKII protein was detected from cell lines expressing FLHKII, S4L-HKII, H5P-HKII and A8L-HKII. A greater than 4-fold decrease in H5P-HKII protein levels as compared with wild-type HKII protein levels was found in the cytoplasmic fraction. The results from the point mutations indicate that the basic, hydrophilic histidine residue at position 5 is critical for HKII binding to the MOM.

## DISCUSSION

There are four isoenzymes of hexokinase in mammalian cell lines. Types I, II, III and IV with types I–III expressed as 100 kDa proteins and thought to have been derived from a gene duplication/fusion of an ancestral 50 kDa hexokinase [16]. Type IV hexokinase is a 50 kDa protein commonly referred to as glucokinase. In addition, HKII has two catalytic sites as compared with one site on types I, III and IV [17,18]. Early studies have shown that hexokinase I and II bind to a MOM pore-forming protein and have referred to the protein as VDAC or mitochondrial porin [19–23]. Subsequent studies have identified the N-terminal domain of hexokinase I and II as necessary for binding to VDAC [24–26].

Using a peptide corresponding to the N-terminal domain for HKII, Pastorino et al. [27] has shown that the first 15 amino acids were important for binding to the MOM. We have presented evidence that the fifth amino acid, histidine, on the N-terminal end of HKII is an important component of the binding site to VDAC. Converting the positively charged, hydrophilic histidine to an uncharged, hydrophobic proline completely prevented the binding of HKII to the MOM. Studies of hexokinase I by the Honzatko laboratory have shown that mutating the fifth amino acid, glutamine to proline (Q5P) resulted in a 10-fold decrease in binding to the mitochondrial membrane [28].

When hexokinase is bound to the MOM it is not a substrate for the ubiquitin system, however unbound hexokinase protein was shown to be a target for the ubiquitination pathway and rapidly degraded [29,30]. We hypothesize that the reduced levels of mutant hexokinase protein found in the cytoplasm are due to ubiquitination but further studies are necessary to determine the fate of the mutant proteins.

Mutating the fourth amino acid from the uncharged, polar, hydrophilic serine to an uncharged, hydrophobic leucine has shown an increased stability of the mutant protein by showing higher protein levels found in the cytoplasm and mitochondrial fractions. The S4L-HKII mutant protein had greater than 6-fold affinity for the mitochondrial membrane as compared with FLHKII. We have made double-mutants of the fourth and fifth amino acids of HKII protein (S4L-H5P-HKII) and have shown that the double-mutant HKII protein maintains the ability to bind to the MOM (unpublished data).

Since mutating of the fourth amino acid enhanced the binding of HKII to the MOM, the relevance to the development of cancers needs to be further investigated. Hexokinase type II was shown to be overexpressed in tumour cells, conferring a 100-fold increase in enzymatic activity relative to normal cells [31]. Subcellular studies have shown that HKII is catalytically active when bound to the MOM, giving HKII preferential access to mitochondrial generated ATP [4,5]. Studies have shown that when HKII expression is down-regulated by siRNA or HKII is prevented from binding to the VDAC on the MOM, the tumour cells are sensitized to chemotherapy agents [6–8,32] and radiation [9]. Alternately, the phosphorylation of VDAC by glycogen synthase kinase 3β (GSK3β) disrupted the ability of HKII to bind to VDAC, potentiating the doxorubicin- and paclitaxel-induced cell death [6].

Using a structure-based virtual screen, we propose to identify drug-like small molecules with a putative mechanism-of-action of translocating HKII from the MOM to the cytoplasm. The results from the present study have identified the histidine located at the fifth position of the N-terminal end of HKII to be critical for binding to the MOM. To pursue a molecular docking screen for the binding site, the complete crystal structure of HKII must be obtained. The Toronto Structural Genomics Consortium (TSGC) identified the crystal structure of HKII [33], however the crystal structure was missing the first 16 N-terminal amino acids. The crystal structures of human hexokinase I are missing the first 10 or 15 amino acids on the N-terminal end [34,35]. The high-resolution crystal structure of yeast HKII does maintain the N-terminal end and the secondary structure was described as an alpha-helix (residues 1–12) and a six-stranded mixed β-sheet (residues 13–76) [36]. The hydrophobic N-terminal region of human HKII has been described as important for binding to the MOM [11,13,37]. We present evidence that the fifth amino acid, histidine, located in the N-terminal domain of HKII is critical for binding to the MOM. Upon crystallization of the complete HKII enzyme, the secondary structure surrounding the histidine can be analysed by structure-based virtual screen to identify putative drug-like compounds that may interfere with the binding of HKII to VDAC on the MOM.

## Abbreviations

aa: amino acid
BAX: BCL2-associated X protein
CIAP: calf intestinal alkaline phosphatase
FLHKII: full-length HKII
GFP: green fluorescent protein
HKII: hexokinase II
MOM: mitochondrial outer membrane
PCR: polymerase chain reaction
PET: positron emission tomography
siRNA: small interfering RNA
VDAC: voltage-dependent anion channel
